# Parasite Recognition and Signaling Mechanisms in Innate Immune Responses to Malaria

**DOI:** 10.3389/fimmu.2018.03006

**Published:** 2018-12-19

**Authors:** D. Channe Gowda, Xianzhu Wu

**Affiliations:** Department of Biochemistry and Molecular Biology, The Pennsylvania State University College of Medicine, Hershey, PA, United States

**Keywords:** malaria, immunostimulatory factors, host receptors, signaling mechanisms, innate immune responses, protective immunity, pathogenesis

## Abstract

Malaria caused by the *Plasmodium* family of parasites, especially *P*.*falciparum* and *P. vivax*, is a major health problem in many countries in the tropical and subtropical regions of the world. The disease presents a wide array of systemic clinical conditions and several life-threatening organ pathologies, including the dreaded cerebral malaria. Like many other infectious diseases, malaria is an inflammatory response-driven disease, and positive outcomes to infection depend on finely tuned regulation of immune responses that efficiently clear parasites and allow protective immunity to develop. Immune responses initiated by the innate immune system in response to parasites play key roles both in protective immunity development and pathogenesis. Initial pro-inflammatory responses are essential for clearing infection by promoting appropriate cell-mediated and humoral immunity. However, elevated and prolonged pro-inflammatory responses owing to inappropriate cellular programming contribute to disease conditions. A comprehensive knowledge of the molecular and cellular mechanisms that initiate immune responses and how these responses contribute to protective immunity development or pathogenesis is important for developing effective therapeutics and/or a vaccine. Historically, in efforts to develop a vaccine, immunity to malaria was extensively studied in the context of identifying protective humoral responses, targeting proteins involved in parasite invasion or clearance. The innate immune response was thought to be non-specific. However, during the past two decades, there has been a significant progress in understanding the molecular and cellular mechanisms of host-parasite interactions and the associated signaling in immune responses to malaria. Malaria infection occurs at two stages, initially in the liver through the bite of a mosquito, carrying sporozoites, and subsequently, in the blood through the invasion of red blood cells by merozoites released from the infected hepatocytes. Soon after infection, both the liver and blood stage parasites are sensed by various receptors of the host innate immune system resulting in the activation of signaling pathways and production of cytokines and chemokines. These immune responses play crucial roles in clearing parasites and regulating adaptive immunity. Here, we summarize the knowledge on molecular mechanisms that underlie the innate immune responses to malaria infection.

## Background

Malaria is a widespread infectious disease that is prevalent in most tropical regions of the world (1–4). About half of the world population is at risk of contracting malaria. World Health Organization has reported an estimated 216 million malaria clinical cases and about 445,000 deaths during 2016 (1). Besides huge health burden and mortality, malaria morbidity is a substantial hindrance to socio-economic development in endemic areas due to the loss of man power (5, 6). The disease is caused by protozoan parasites of the genus *Plasmodium*. Five parasite species infect humans that include *Plasmodium falciparum, P. vivax, P. malariae, P. ovale*, and *P. knowlesi* (7–9). However, most malaria infections are caused by *P. falciparum* and *P. vivax*, and infections by the other three species are relatively rare. Several parasite species, including *P. berghei, P. yoelii, P. chabaudi*, and *P. vinckei*, infect rodents, but not humans (10). Different strains of rodent parasites used in laboratories show distinct growth rates, and infected mice exhibit a range of immunological and pathological conditions, resembling a wide spectrum of pathophysiologic conditions of human malaria infection. As such, mice infected with different parasite strains are useful models to study distinct systemic and organ-specific clinical conditions of human malaria.

Malaria is a highly complex disease that displays a wide variety of pathological conditions. At the early stages, malaria infection presents a number of systemic clinical conditions, including the characteristic periodic fever, chills, headache, dizziness, malaise, abdominal discomfort, nausea, and muscle and joint aches (11, 12). As the infection progresses and parasite biomass increases, pathogenic processes follow, resulting in severe anemia, blood acidosis, splenomegaly and hepatomegaly, acute respiratory distress syndrome, and several other clinical conditions. In the case of *P. falciparum*, the infected red blood cells (IRBCs) bind to certain cell surface proteins of vascular endothelia, including CD36, intracellular adhesion molecule 1 (ICAM-1), vascular adhesion molecule 1 (VCAM-1), and endothelial protein receptor (EPCR) (4, 13–20). These binding events allow parasites to sequester in organs, such as brain, lungs, liver, intestine, dermal tissues, and placenta, thereby avoiding splenic clearance. Parasite sequestration contributes to single and multiorgan fatal pathologic conditions, including cerebral malaria, and renal, liver and lung dysfunction and failure. In pregnant women, *P. falciparum* sequesters in the placenta through the binding of IRBCs to chondroitin 4-sulfate in the intervillous space and on the syncytiotrophoblast cell surface (21–23). This process contributes to pregnancy-associated malaria, characterized by a number of clinical conditions, including low birth weight, abortion, and death in the baby and the mother (24). The binding of IRBCs to the endothelial cell surface proteins in the microvasculature of vital organs and chondroitin 4-sulfate in the placenta is mediated by a family of antigenically variant parasite proteins encoded by about 60 *var* genes (15, 16, 25). These proteins are collectively called *P. falciparum* erythrocyte membrane protein 1 (PfEMP1). PfEMP1 confers virulence to *P. falciparum* through IRBC binding to endothelial cells in various organs contributing to microvascular plugging and hypoxia. In addition, IRBC-binding and parasite accumulation amplify inflammatory responses locally, leading to immune cell infiltration, endothelial damage, and organ dysfunction and failure. *P. vivax* lacks PfEMP1 ortholog (26) and thus, *P. vivax* is less virulent compared to *P. falciparum*. However, in recent years, *P. vivax* is becoming increasingly virulent, causing severe malaria in significant numbers (11, 27–30). Although, the underlying reasons in *P. vivax* causing severe illnesses remain unclear, drug resistance and changes in genetic makeup appear to be significant factors (31, 32).

Innate immune responses that are initiated in response to malaria infection play key roles both in the development of protective immunity and pathogenesis (14, 33–38). Early pro-inflammatory responses regulate antiparasitic Th1 development and promote effector cell function for efficiently clearing infections. Usually, as infection progresses, pro-inflammatory responses are gradually downregulated with parallel increase in anti-inflammatory responses (39). Generally, this leads to Th2 development, resulting in balanced pro-/anti-inflammatory and Th1/Th2 responses and resistance against pathogenesis (35, 39–42). However, this is not always the case. Depending upon the host-parasite interaction dynamics, factors such as parasite sequestration and alterations in host genetics, pro-inflammatory responses may be overly upregulated, resulting in systemic and organ-related severe illnesses.

During the past two decades, there has been a substantial progress in our understanding of the host receptors that sense parasite factors involved in inducing immune responses and the associated signaling pathways (43–47). A substantial progress has also been made in our understanding of parasite immunostimulatory factors that are the targets of host receptors (43–47). However, much remained to be learned on the spectrum of molecular and cellular processes and immune responses that are initiated upon parasite sensing. Development of protective immunity to malaria requires repetitive infection over a period of time (48, 49). Information is limited as to whether and how the innate immune responses contribute to the inefficient development of protective immunity to malaria. In addition to signaling initiated by specific sensing of parasite factors, signals induced by processes such as phagocytosis and cytoadherence also contribute; information is also limited as to whether and to what extent these signaling contribute to the overall innate immune responses. In this review, we are providing an overview of the available information on the molecular and cellular mechanisms of parasite-host interactions that involved in innate immune responses to malaria.

## Malaria Infection

Malaria infection begins with the entry of sporozoite form of parasites when infected mosquitoes inject saliva during blood meal (7, 46, 50–52). A substantial number of injected sporozoites is unable to enter the blood stream and stuck in the dermis, and these parasites are removed likely by the resident macrophages (Mφs). Those sporozoites that enter the blood stream target liver, where they exclusively infect hepatocytes. In hepatocytes, parasites reside inside parasitophorous vacuole formed during invasion and develop into merozoites over a period of 7–10 days in humans and 2 days in rodents, vastly expanding hepatocyte size. Upon parasite maturation, each infected hepatocyte releases 10,000–30,000 merozoites into the blood stream (46, 50–52). This period of cell cycles represents the first stage of malaria infection, referred to as the liver stage or the tissue stage infection. The merozoites released from the matured infected hepatocytes are called exo-erythrocytic merozoites. These merozoites do not infect hepatocytes, but instead exclusively invade red blood cells, and reside inside parasitophorous vacuole. Soon after invasion, parasites appear morphologically like rings inside IRBCs. Parasites then develop into early and late trophozoites, and finally undergo schizogony to form differentiated merozoites, which are released into the blood stream. Each matured erythrocytic stage schizont releases 8–24 merozoites (53) (Table 1), which can invade red blood cells. The parasite developmental process inside red blood cells occurs over a period of 24–72 h, depending on the *Plasmodium* species (Table 1). This cycle of invading red blood cells and parasite growth is called the blood stage infection. The repetitive erythrocytic cell cycles result in the exponential growth of parasites and if the growth is unchecked, most red blood cells are consumed, resulting in severe anemia and pathologies, and death. However, soon after infection, the innate immune system detects parasites at both the liver and blood stages, and responds by inducing pro-inflammatory cytokines and chemokines. The cytokines prime phagocytes for an efficient uptake and clearance, while chemokines help recruit effector cells to sites, where parasites are sequestered or accumulated, for effective infection clearance.

**Table 1:** Prevalence and features of human blood stage malaria parasites.

**Parasite species**	**Prevalence**	**Erythrocyte stage life cycle duration**	**Merozoite numbers in matured IRBCs**	**Cytoadherence capacity and organs where adherence occurs**	**Frequency of causing severe illnesses and fatality**	**References**
*P. falciparum*	Worldwide (high prevalence in Africa)	48 h	8–24	Strong; in most organs, including skin, intestine and placenta	Often causes severe illnesses, highly fatal	(53)
*P. vivax*	Asia, Latin America, some parts of Africa	48 h	12–18	Weak; mostly in lungs	Mostly uncomplicated malaria, occasionally fatal	(53)
*P. ovale*	Africa, Western pacific islands	48 h	8–16	Absent	Mostly uncomplicated malaria, fatality is rare	(53, 54)
*P. malariae*	Worldwide	72 h	6–12	Absent	Mostly uncomplicated malaria, fatality is rare	(53, 54)
*P. knowlesi*	Southeast Asia	24 h	Up to 16	Weak; in lungs, and likely brain and other organs	Uncomplicated to severe illnesses (60–70% of infected cases develop ARDS^a^)	(8, 9, 55)

a *ARDS, acute respiratory distress syndrome*.

## Pathogen Sensing Mechanisms

Hosts respond to various infections by sensing certain evolutionarily conserved molecules (signature structures) of pathogens called pathogen-associated molecular patterns (PAMPs); these include bacterial LPS and peptidoglycan, fungal glucans, and microbial DNA and RNA, and even self-DNA under certain pathologic conditions (56–61). Host detects PAMPs through receptors called pathogen-recognition receptors (PRRs). Host also detects certain endogenous factors released during infection and thus can induce danger signaling. These factors are called danger-associated molecular patterns (DAMPs). Examples of DAMPs include high mobility box 1 (HMGB1), HSP70, SP100 family of proteins, and degraded hyaluronic acid (62–65). The innate immune system is equipped with a wide range of PAMP- and DAMP-recognizing PRRs. After recognition of PAMPs and DAMPs by PRRs, the innate immune cells are activated through the initiation of specific signaling pathways, producing cytokines and chemokines. PRRs are present at various cellular locations, including outer surface of plasma membrane, luminal surface of endosomal membrane, outer membrane of mitochondria, and cytosol (59, 60, 66). Prominent among transmembrane PRRs are toll-like receptors (TLRs) (56–59, 61); others transmembrane PRRs include, c-type lectin receptors, such as mannose- and galactose-binding proteins, and scavenger receptors, such as CD36, CD204, and MARCO (67). Examples of cytosolic sensors include dectin-1 that binds fungal β-1,3-glucan, cyclic GMP-AMP synthase (cGAS, senses dsDNA), retinoic acid-inducible gene-I (RIG-I, respond to viral RNA), RIG-I-like receptors (RLRs), such as melanoma differentiation-associated gene 5 (MDA5) that respond to dsRNA, nucleotide-binding oligomerization domain (NOD)-like receptors (NLRs, senses bacterial peptidoglycan), and absent in melanoma 2 (AIM2, respond to dsDNA). Malaria parasites are sensed by several receptors, leading to cell activation and immune responses (see below). Several excellent reviewers that comprehensively discuss various host-pathogen interactions and signaling mechanisms are available (56–60, 62–66, 68–70).

## Malarial PAMPs and Host PRRs

A notable feature of malaria infection is that the liver stage is clinically completely silent, that is, the host does not exhibit any symptoms of malaria (71–73). All the malaria clinical conditions and fatal illnesses are manifested during the blood stage infection (74). At both stages of infection, the host detects parasites immediately after infection and initiates innate immune responses. These responses are geared toward clearing the infection and shaping the development of protective adaptive immunity (14, 34, 44, 71–78). However, the complex parasite-host interaction dynamics are not always in favor of achieving this goal, but instead often result in dysregulated immune responses and uncontrolled parasite growth, leading to pathogenesis. Understanding the malaria parasite-host interaction dynamics that shape the parasite-specific immunity and the molecular and cellular processes that contribute to pathogenesis is important for developing suitable treatment strategies. Below, we summarize the advancements that have been made in identifying the malaria PAMPs, the cognate host PRRs, and the signaling events that lead to innate immune responses.

## Liver Stage Parasite Sensing

**Figure 1:**
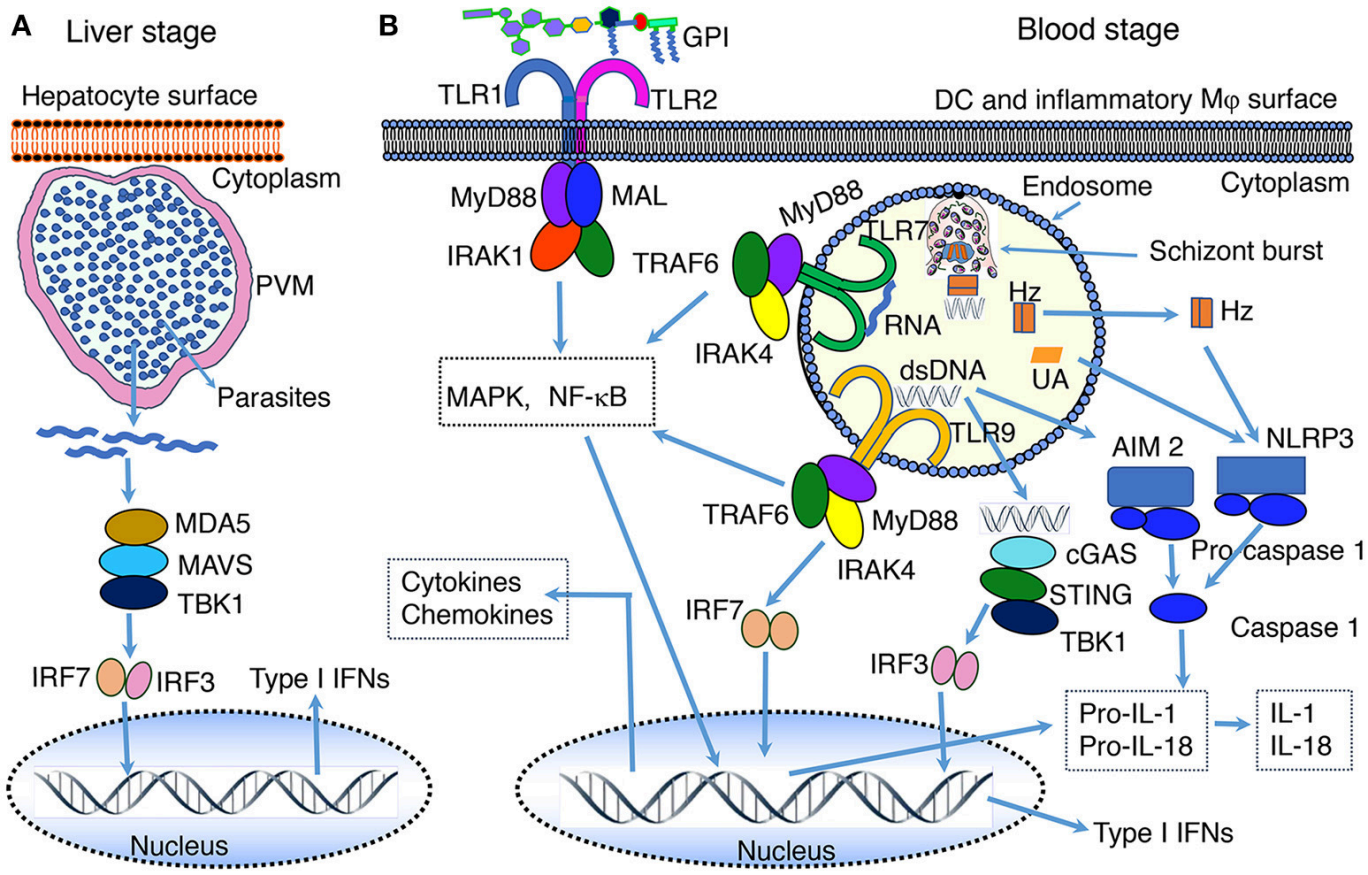
PAMP-PRR interaction-induced signaling pathways. **(A)** RNA of the liver stage parasites growing inside hepatocytes is recognized by MDA5 leading to the activation of MAVS-TBK1-IRF3/IRF7 signaling and downstream production of type I IFNs. **(B)** At the blood stage infection, parasite DNA, RNA and GPI interact with, respectively, TLR9, TLR7, and TLR2, leading to the activation of primarily MAPK and NF-κB signaling pathways and downstream cytokine and chemokine responses. In the cytosol, similar to the liver stage parasite RNA, the blood stage parasite RNA is sensed by MDA5 leading to the activation of MAVS-TBK1-IRF3/IRF7 signaling (see **A**). However, this signaling seems induce the expression of SOCS1, which downregulate RNA-TLR7-induced type I IFN production (81). Parasite DNA in the cytosol is sensed by cGAS, resulting in the activation of STING-TBK1-IRF3 signaling and type I IFN response. Parasite DNA also activates AIM2 inflammasome, which cleaves pro-caspase 1 to activate caspase 1. Hemozoin (Hz) and uric acid (UA) induce danger signaling, activating NLRP3 inflammasome and the cleavage of pro-caspase 1 to activate caspase 1. Parasites have also been reported to activate NLRP12 inflammasome through unidentified interaction, leading to the cleavage of pro-caspase 1 to activate caspase 1 (44, 82). It appears that microparticles released from IRBCs and heme produced during infection activate TLR4 signaling (83, 84). Ligands bind to TLR4 homodimer through the cooperation of accessory proteins CD14 and MD2, leading to MAPK, NF-κB and TRIF-TBK1-IRF3 signaling. Note: the diagram depicts a simplified version of indicated signaling pathways and additional details can be found in review articles (44, 56–70). The abbreviations are defined in footnote in page 1.

Since the liver stage malaria infection is clinically silent, it has long been thought that parasites inside hepatocytes grow undetected by the innate immune system. However, recent studies in *P. berghei*- and *P. yoelii*-infected mice show that, although parasites inside hepatocytes are shielded from recognition by Mφs and dendritic cells (DCs), the growing parasites are recognized by the cytosolic PRRs of hepatocytes, initiating antiparasitic type I IFN response (79, 80). The parasites in infected hepatocytes are detected through the interaction of parasite RNA with RIG-I family of proteins homolog called melanoma differentiation-associated protein 5 (MDA5), but not by RIG-I, leading to the activation of MDA5-MAVS-IRF3/IRF7 signaling axis and downstream production of type I IFN (79, 80) (Figure 1). This signaling event results in an array of type I IFN receptor (IFNαR)-mediated innate immune responses, which include: (i) expression of interferon-stimulated genes by hepatocytes; (ii) production of chemokines by hepatocytes, and chemotaxis-mediated recruitment of Mφs, neutrophils and lymphocytes to the proximity of infected hepatocytes; (iii) production of IFN-γ and chemokines by NK and NKT cells, which are abundantly present in the liver; (iv) infiltration of NK and NKT cells to the liver; (v) CD1d-resitrcted elimination of infected hepatocytes by NKT cells (79, 80). Since parasites reside inside parasitophorous vacuole, it appears that parasite RNA is exported to the cytosol, but not to phagolysosomes. It is unlikely that cytosolic RNA can enter endosomes and moreover hepatocytes may not express significant levels of TLR7. Thus, it appears that cytosolic sensors are the only PRRs that interact with parasite factors in infected hepatocytes. These findings represent a significant advancement in our understanding of parasite recognition mechanisms involved in innate immune responses to the liver stage malaria infection. However, it remains unclear if parasite DNA is exported to the cytosol, where it can be sensed by cytosolic nucleic acid sensors. DNA is a prominent immunostimulatory PAMP of the blood stage malaria parasites and the AT-rich stem loops of parasite DNA can induce the production of type I IFN through sensing in the cytosol (44, 60). If the liver stage parasite DNA has no role in type I IFN response, then DNA is either not exported to the cytosol of hepatocytes or non-stimulatory.

## Blood Stage Parasite Sensing

As mentioned earlier, the blood stage infection accounts for all the pathological conditions of malaria (74). In infected non-immune people, *P. falciparum* parasites multiply rapidly through the release of large numbers of merozoites by matured schizonts to the blood circulation every 48 h (53, 85). Some of the released merozoites invade erythrocytes and the reminders become dead and are likely targeted by Mφs and DCs. In addition, the burst of schizont stage erythrocytes releases large amounts of parasite's digestive vacuoles containing hemozoin and other waste products. The blood stage parasites grow synchronously by adapting to daily rhythm of systemic TNF-α production and the level of glucose in the blood (86). Thus, parasites develop at similar rates, releasing merozoites, digestive vacuoles and other contents more or less at the same time point (86). This leads to peak concentrations of parasite stimulatory components, to which the innate immune system potently responds and induces the production of pro-inflammatory cytokines and chemokines at high levels. This periodic elicitation of strong inflammatory responses within a narrow window of time period after each erythrocytic cell cycle is responsible for the characteristic periodicity of malaria paroxysms, including periodic recurrent of peak levels of fever, chills, headache, shock, and malaise. Several malaria parasite PAMPs have been identified, and the mechanisms by which they are sensed and the associated signaling pathways and immune responses have been delineated (44, 46, 73, 87). This body of information represents a substantial progress in our understanding of malaria innate immunity. Below we describe the current knowledge on malaria parasite PAMP and host PRR interaction mechanisms involved in innate immune responses.

## Malarial PAMPs

### GPI

*P. falciparum* glycosylphosphatidylinositol (GPI) is the first factor that was identified as a malaria parasite PAMP (88). Structurally, parasite GPI comprises a heterogenous group of molecules consisting of triacylated phosphatidylinositol linked to the glucosamine moiety of glycan having four mannose residues and a glucosamine residue (89–91). The structural heterogeneity of malaria GPI is due to the variation in length and level of unsaturation in acyl residues present at different positions of the phosphatidylinositol moiety. This structural heterogeneity in the lipid moieties has no bearing on immune response-inducing activity of GPI. This is evident from the fact that the *sn-2 lyso* GPI, obtained by the removal of acyl moiety at the *sn-2* position of parasite GPI, could efficiently induce cytokine responses similar to the unmodified parasite GPI. The biosynthesis of GPI is essential for the survival of parasites as GPI anchors several proteins of merozoites to the plasma membrane that are involved in erythrocyte invasion (92–94). In the absence of GPI anchoring, these proteins are not expressed on the surface and hence merozoites cannot invade erythrocytes. Malaria parasites synthesize GPI in several folds excess over the actual amounts needed for anchoring proteins to the surface of merozoites and thus, significant amounts of GPI remain not linked to proteins (90). The GPI molecules that are not linked to proteins are exposed on the cell surface and thus are likely targeted by the innate immune system.

The identification that parasite GPI is a malaria inflammatory response-inducing pathogenicity factor was based on the observation that the purified parasite GPI could induce strong pro-inflammatory responses by Mφs (88). When administered to mice, GPI induced symptoms that resembled the systemic clinical conditions of malaria, including pyrexia, cachexia, hypoglycemia, and TNF-α-induced sepsis. In several subsequent studies, GPI was shown to induce a wide range of immune responses, including the production of TNF-α and IL-1 by Mφs, expression of nitric oxide synthase by Mφs and endothelial cells, and the expression of ICAM-1, VCAM-1, and E-selectin by leukocytes and endothelial cells through the activation of several signaling events (95–97). Consistent with the property of GPI in inducing malaria-like symptoms, immunization of mice with a synthetic glycan portion of GPI produced anti-GPI antibodies, and immunized mice infected with *P. berghei* ANKA, an experimental cerebral malaria model, were protected from cerebral malaria (98). Further, the presence of anti-GPI antibodies in people in malaria endemic was associated with a significant protective immunity against malaria illnesses (99, 100).

Subsequent studies have shown that *P. falciparum* GPI activates Mφs through the induction of an outside-in signaling, without binding to plasma membrane or being internalized by the cells, but instead by recognition through weak interactions on the cell surface (101). The intact structure of GPI is essential for bioactivity as neither the carbohydrate moiety nor the triacylated phosphatidylinositol lipid portion can induce immune responses. Further studies have shown that the parasite GPI induced production of pro-inflammatory cytokines by Mφs occurs through recognition mainly by TLR2-TLR1 heterodimer and to a much lesser extent by TLR4 (102–105); Figure 1 and Table 2. Sensing of GPI by TLR2-TLR1 leads to the activation of ERK, p38, JNK MAPK, and NF-κB signaling pathways, which differentially contribute to the production of various inflammatory mediators (103). Thus, malaria parasite GPI is mainly a TLR2-activating PAMP.

**Table 2:** Innate sensing of malaria parasites and signaling mechanisms.

**PRRs (host receptors)**	**PRR cellular location**	**PAMPs and DAPMs**	**Signaling pathway**	**References**
**TLRs**				
TLR1,2 dimer	Cell surface	GPI	MAPK, NF-κB	(43, 89, 103, 104)
TLR4	Cell surface	GPI, heme, IRBC micro-particles	MAPK, TRIF, NF-κB	(43, 44, 83, 84)
TLR7	Endosome	RNA	MAPK, NF-κB	(106)
TLR9	Endosome	DNA	MAPK, NF-κB	(44, 107, 108)
**CYTOSOLIC NUCLEIC ACID SENSORS**				
MDA5	Cytosol	RNA	MAVS-TBK1-IRF3/IRF7	(79–81)
cGAS	Cytosol	DNA	STING-TBK1-IRF3/IRF7	(44, 109, 110)
AIM2	Cytosol	DNA	NLRP3 inflammasome	(44, 109, 111)
**DANGER SIGNALING**				
-	Cytosol	Hemozoin	NLRP3 inflammasome	(45, 111–114)
-	Cytosol	Uric acid	NLRP3 inflammasome	(114–116)
-	Cytosol	Unidentified factor	NLRP12 and NLRP4 inflammasome	(44, 82)
**PHAGOCYTIC RECEPTORS**				
CD36	Cell surface	PfEMP1, and unidentified ligand(s)	Src/Syk, MAPK	(117–120)
**ADHESION RECEPTOR**				
ICAM-1	Cell surface	PfEMP1	Src-PI3K-Akt, Rho, MAPK?	(121, 122)
EPCR	Cell surface	PfEMP1	Not known

### DNA

A large body of accumulated data over the past two decades demonstrates that microbial (bacteria, viruses, and parasites) DNA, and self-DNA in some pathological situations, function as PAMP and are recognized by TLR9 in endosomes and by DNA sensors in the cytosol (56–60). In the case of malaria, the first demonstration that TLR9 senses malaria parasites and induces immune responses was by Pinchyangukul et al. (123). They showed that a soluble component in the schizont extract of *P. falciparum* that was heat labile and precipitable with ammonium sulfate (the description agrees with the active component being protein-DNA complex) induces cytokine and chemokine responses by human plasmacytoid DCs (pDCs) and mouse DCs through the activation of TLR9-MyD88 signaling pathway. Subsequently, it was shown that the TLR9 signaling-inducing malaria factor is DNA (107, 108, 124). TLR9 specifically recognizes the unmethylated CpG motifs of DNA (68, 69). The genomic DNA of *P. falciparum* and *P. vivax* contain, respectively, ~300 and ~2,000 CpG motifs (109). The higher content of CpG motifs is likely responsible for the strong fever-inducing ability of *P. vivax* compared to *P. falciparum* (125).

Malaria parasite DNA enters the endosomes of the innate immune cells, such as Mφs and DCs, through phagocytic uptake of IRBCs, merozoites, the nuclear material of parasites, DNA-protein-hemozoin complex, and DNA-containing immune complexes (107, 108, 124, 126). The endosomes are then fused to lysosomes to form phagolysosomes. In the acidic environment of phagolysosomes, DNA is released and recognized by TLR9, leading to the activation of MAPK and NF-κB signaling pathways and cytokine and chemokine responses (Figure 1 and Table 2). In addition to TLR9, parasite DNA is recognized by cytosolic DNA sensors upon the release of phagolysosomal contents into the cytosol. In the cytosol, several distinct cytosolic sensors can potentially recognize DNA (Figure 1). Thus far, it has been demonstrated that two cytosolic PRRs sense parasite DNA: (i) cGAS sensing dsDNA and inducing the activation of STING-TBK1-IRF3 signaling and downstream production of type I IFNs (44, 110), and (ii) AIM2 recognizing dsDNA, resulting in the activation of inflammasome and caspase 1. The activated caspase 1 converts pro forms of IL-1 and IL-18 into active IL-1 and IL-18. Robust production of IL-1 and IL-18 in response to malaria infection requires, in addition to inflammasome signaling, TLR (mainly TLR9 and TLR7) mediated production of pro-IL-1 and pro-IL-18. *P. falciparum* genomic DNA contains >80% AT nucleotides. The AT-rich motifs of parasite DNA form loop structures, which have been shown to induce type I IFN response through STING-TBK1-IRF3 signaling (109). In some viruses, the AT-rich loop motifs of DNA are transcribed by RNA polymerase III to form double-stranded RNA containing 5′-triphosphate that induces type I IFNs through RIG-I-MAVS-IRF3 signaling (110). However, in the case of *P. falciparum* DNA, pol III-dependent recognition of AT-rich motifs of DNA seems to be not involved in cytosolic sensing as deficiency in pol III had no effect on immune responses to the parasite DNA (109).

### RNA

The innate immune system senses RNA of both the liver and the blood stage parasites. In the liver stage, as described in the section Liver Stage Parasite Sensing above, RNA is recognized exclusively in the cytosol by MDA5 (Figure 1) (79, 80). In contrast, in the blood stage infection, mouse parasite RNA is recognized by TLR7 in phagolysosomes of DCs, leading to type I IFN production (Figure 1). In fact, studies in a mouse model of *P. chabaudi-*infection showed that type I IFN production is the earliest cytokine response (24 h postinfection) during the blood stage malaria infection (127). Parasite RNA enters endosomes of Mφs and DCs upon the uptake of parasites/parasite components, inducing type I IFN response through TLR7 signaling (81, 106, 128). Type I IFNs thus produced prominently contribute to the upregulation of pro-inflammatory cytokines, such as IFN-γ and IL-12, during the early stages of infection (106). RNA of the blood stage mouse parasites is also sensed by cytosolic MDA5, leading to type I IFN production (81).

While it is clear that RNA induced TLR7 signaling plays an important role in early type I IFN production in mouse malaria, it remains unclear whether or to what extent RNA of human parasites is able to induce type I IFN response. This is because, although RNA of human malaria parasite *P. falciparum* has been reported to induce cytokine responses through TLR7 recognition, the reported activity appears to be very low (106). Further studies are needed to determine whether or to what extent *P. falciparum* RNA is immunostimulatory.

## Malarial Danger-associated Molecular Patterns (DAMPs)

### Hemozoin

Hemozoin is a hydrophobic, crystalline insoluble polymer of heme formed during the digestion of hemoglobin by parasites in the digestive vacuoles to use the released amino acid as a food source (45, 129–131). In parasites, hemozoin is associated with lipids and proteins, and is released into the blood circulation when merozoites are egressed from matured schizonts. Hemozoin by itself is an inert material and appears to have no specific receptor for recognition, but it influences the innate immune responses to malaria parasites in several ways; reviewed in Olivier et al. (45). Studies have reported that hemozoin is a carrier of malaria parasite DNA into endosomes for TLR9 recognition (107). Although how and where parasite DNA associates with hemozoin have not been specifically studied *in vivo*, there exist several possibilities. Malaria merozoites egressed from the matured schizonts have a half-life of <5 min (132), and many merozoites cannot invade red blood cells within this short period time. These uninvaded merozoites are likely to be lysed, releasing DNA that may complex with hemozoin *via* the associated proteins. Additionally, Mφs and neutrophils undergo apoptotic death after ingesting IRBCs (133), releasing the degraded parasite materials, including DNA, RNA, hemozoin and other components. The released DNA can complex with hemozoin. However, hemozoin is not obligatory for the entry of parasite DNA into endosomes. The parasite nuclear material, DNA containing immune complexes, merozoites, and IRBCs are also taken up by phagocytic cells (107, 108, 124, 126). It is difficult to quantify *in vivo* the extent to which DNA enters endosomes through phagocytic uptake of infected erythrocytes, merozoites and nuclear materials compared to hemozoin-bound DNA in inducing immune responses.

Since hemozoin is an inert, indigestible material, it curtails the ability of Mφs to induce innate immune responses to malaria. Upon uptake of IRBCs or hemozoin, Mφs become immunosuppressive and dysfunctional due to damages caused by oxidative burst (134). Monocytes and Mφs that ingest parasite IRBCs undergo apoptotic death with little or negligible release of cytokines, although cytokines are expressed to certain extent (133). Phagocytic internalization of large amounts of whole parasites and non-digestible hemozoin renders Mφs nonfunctional because of phagolysosomal acidification and apoptotic death (133, 135). Hemozoin is known to inhibit the differentiation and maturation of human monocyte-derived DCs as well (136, 137). However, certain subsets of inflammatory Mφs, such as spleen red pulp Mφs, CD169^+^ Mφs, and CD16^+^ monocytes produce cytokines in response to malaria parasites (126–128). It is not known whether hemozoin significantly alters the capacity of these cells to produce cytokines and chemokines.

The effect of hemozoin on DC function remains unclear, reviewed in Wykes and Good (138). A previous study has shown that hemozoin-internalized DCs localized in T cell areas of the spleen in *P. chabaudi*-infected mice and that T cell activated by these DCs lacked effector function (139). These results suggest that hemozoin considerably compromises DC function. However, in a later study, DCs efficiently produced DNA-TLR9 signaling-induced cytokines in response to DNA bound to natural hemozoin and thus it was suggested that hemozoin enhances the activity of DNA by facilitating its entry to endosomes (107). It remains unclear whether this is the case in infected host. On the other hand, it has been shown that subsequent to the uptake of parasite components and the activation of TLR7 and TLR9 signaling, hemozoin destabilizes phagolysosomal membrane, leading to the release of nucleic acid and hemozoin into the cytosol (111). The released parasite components are sensed by cytosolic PRRs, leading to several signaling pathways (44, 81, 111–113), including: (i) cGAS-STING-TBK1-IRF3 signaling by dsDNA; (ii) MDA5-MAVS-TBK1-IRF3 signaling by RNA; (iii) STING signaling mediated by AT-rich motifs through sensing by an unidentified receptor; (iv) AIM2 inflammasome signaling induced by parasite DNA, and (v) NLRP12 inflammasome activation *via* an unidentified mechanism (82). In addition, hemozoin also activates NLRP3 inflammasome (45, 111–113); Figure 1 and Table 2. The activation of NLRP3 inflammasome by malarial hemozoin is mediated through the activation of Lyn and Syk tyrosine kinases (45). Synthetic hemozoin also activates Lyn and Syk signaling and, depending on the morphology and particle size, induces distinct immune response. It appears that hemozoin has no specific PRR, but it induces a danger signal (112, 113); hence, hemozoin is a DAPM. While nucleic acid-TLR-mediated signaling results in the synthesis of large amounts of pro-IL-1, inflammasome signaling and activation of caspase 1 lead to the cleavage of pro-IL-1 to the active, fever-inducing IL-1. Thus, hemozoin plays a significant role in the production of IL-1, contributing to fever induction in infected people. Besides hemozoin, parasite biomass, and purified merozoites that are devoid of hemozoin can potentially activate inflammasome and caspase 1 as it is known that even inert materials such as alum, asbestos, silica, uric acid crystals and cholesterol particles, activate NLRP3 inflammasome (45).

### Uric Acid

Uric acid is the final oxidation product of purine metabolism and is released in large amounts by dying cells. Under physiological conditions, uric acid exists as a monoionic urate and forms insoluble monourate crystal (140). Uric acid is an important antioxidant in plasma and can induce protective anti-inflammatory responses in vascular and other diseases (141, 142). However, excessive formation of uric acid promotes pathogenic conditions, such as severe and chronic inflammatory arthritis, gout, and certain metabolic syndromes (141, 142). The pathogenic role of uric acid is due to the activation of NLRP3 inflammasome, which results in caspase 1 activation and conversion of pro-IL-1 into active IL-1 (114–116); Figure 1 and Table 2. During malaria, a large number of parasite-ingested Mφs, neutrophils and other immune cells die, which likely release high levels of uric acid. Importantly, a significant amount of uric acid is formed during purine nucleotide metabolism by parasites and accumulates as a precipitate in IRBCs (143). Large amounts of hypoxanthine also accumulate in parasite IRBCs. The uric acid precipitate and hypoxanthine are released to the blood upon schizont burst. In the blood stream, hypoxanthine is oxidized to uric acid. In agreement with its pathogenic role, high levels of uric acid are found in the blood circulation of patients having severe malaria (144).

## Phagocytosis- and Adherence-Induced Signaling

Phagocytic uptake of parasite IRBCs, merozoites, hemozoin, and immune complexes by Mφs, neutrophils and DCs is an important process during malaria infection. Studies have shown that phagocytosis generally activates Src/Syk family of nonreceptor kinases, leading to the activation of a wide range of signaling pathways, including PKC, RAS-ERK, ${\text{Ca}}_{2}^{+}$, NF-κB signaling (145–147). These signaling events can integrate into TLR and inflammasome signaling, thereby modulating immune responses (148). Phagocytosis of malaria parasites and hemozoin suppresses immune function of Mφs and monocytes due to phagolysosomal acidification (133, 135). However, phagocytosis by DCs and inflammatory Mφs likely induces Src/Syk-mediated signaling, contributing to immune responses. The signaling induced by CD36-mediated phagocytosis and IRBC adherence in malaria immune responses are described below.

### CD36 Signaling

**Figure 2:**
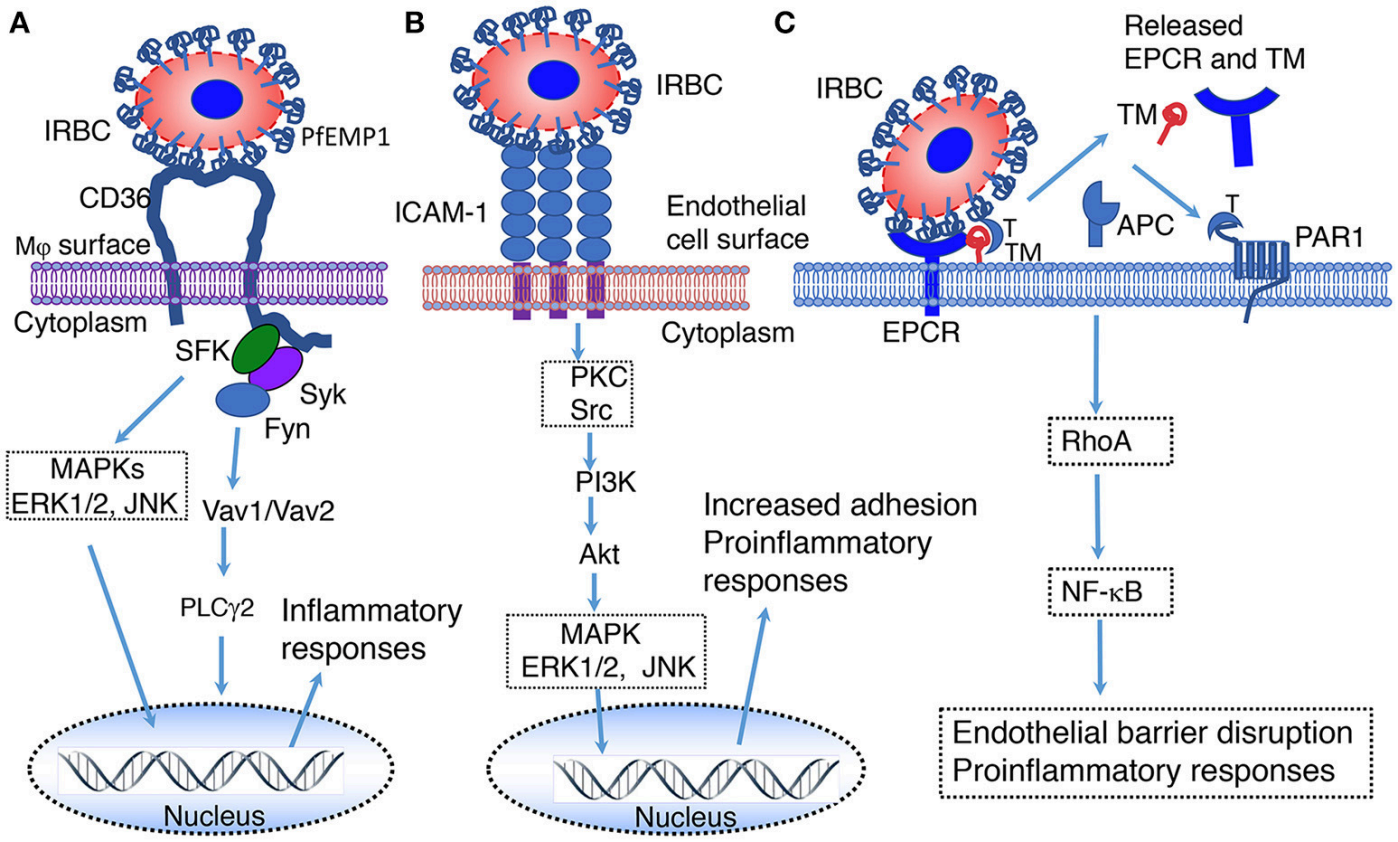
Illustration of predicted pathways activated by the binding of PfEMP 1 expressed on the IRBC surface to CD36 **(A)**, ICAM-1 **(B)** and EPCR **(C)**. **(A)** Interaction of PfEMP1 on the surface of IRBC with CD36 on the surface of macrophages. SFK, Src family of protein tyrosine kinases; Syk, spleen tyrosine kinase; Fyn, proto-oncogene tyrosine protein kinase Fyn; MAPK, mitogen-activated protein kinase; ERK1/2, extracellular signal-regulated kinase 1 and 2; JNK, c-Jun N-terminal kinase; Vav1/Vav2, Vav family of guanine nucleotide exchange factors; PLCγ2, phospholipase Cγ2 (151). **(B)** Interaction of PfEMP1 on the surface of IRBC with ICAM-1 on the surface of endothelial cells surface. PKC, protein kinase C; Src, proto-oncogene tyrosine-protein kinase Src; PI3K, Phosphatidyl inositol 3-kinase; Akt, RAC-alpha serine/threonine-protein kinase (152). **(C)** Interaction of EPCR on the surface of endothelial cells with PfEMP1 on the surface of IRBCs (153–155). EPCR, endothelial protein C receptor; TM, thrombomodulin; T, thrombin; APC, Activated protein C; PAR1, protease-activated receptor 1; RhoA, Ras homolog gene family, member A GTAase.

CD36 is a multifunctional class B scavenger receptor that binds diverse types of ligands and pathogens, facilitating internalization by cells (67, 118, 149). Many cell types, including Mφs, DCs, platelets, and endothelial cells, express CD36. CD36-mediated phagocytosis of pathogens and pathogenic molecules activates Src/Syk kinases, leading to the activation of ERK, p38 and Jun members of the MAPK signaling pathways and NF-κB (67, 118, 119, 150) (Figure 2). Signals of these pathways synergize with TLR and inflammasome signaling, contributing to innate immune responses (119, 156). In malaria, CD36 plays several roles, including (i) mediating the sequestration of *P. falciparum* in vascular capillaries through the binding of adhesive PfEMP1 expressed on the surface of IRBCs, (ii) phagocytic uptake of parasites, and (iii) enhancing innate immune responses (120, 157–162). Studies have shown that CD36-induced MAPK signaling contributes to the production of TNF-α in mouse malaria infection and modulates parasite GPI-induced cytokine responses in mouse Mφs and human blood DCs (120, 161, 163). In *P. falciparum* endemic regions, single nucleotide polymorphisms in the *Cd36* gene have been linked to protection from cerebral and other severe malaria (164, 165). A more recent study in *P. yoelii*-infected mice demonstrated that CD36 significantly contributes to cytokine responses by innate immune cells, and the upregulation of MHCII expression, phagocytic activity, Th1 responses and expression of complement and Fc receptors (117). Overall, these responses lead to decreased parasite burden in infected host. It is possible that, similar to CD36, other scavenger receptors, such as MARCO modulate innate immunity to malaria to a certain extent.

### EPCR Signaling

Endothelial protein C receptor (EPCR), which is also known as activated protein C (APC) receptor, is a transmembrane glycoprotein in vasculatures that binds protein C and promotes thrombin-thrombomodulin complex-mediated protein C activation (166). Under normal conditions, thrombomodulin on the endothelial surface binds thrombin and activates protein C to APC; this process is strongly promoted by EPCR. APC is detached from EPCR and inactivates blood coagulation factors Va and VIIIa, thereby exerting anticoagulant effect. APC binding to EPCR confers cytoprotective roles, such as antiapoptotic, anti-inflammatory and barrier stabilization responses (153–155). In malaria, EPCR binds certain members of PfEMP1 expressed on the surface of IRBCs and contributes to severe malaria (17, 167–169). Although the downstream events of EPCR-PfEMP1 binding-mediated signaling remain to be understood, it appears that the binding results in the loss of EPCR and thrombomodulin from the endothelial cell surface by shedding, leading to decreased protein C activation and compromised EPCR-mediated protection (Figure 2). This may reduce anti-inflammatory responses and increase thrombin production, blood coagulation, pro-inflammatory responses, endothelial cell apoptosis, leading to the loss of barrier function. The cumulative effects of these responses may contribute to endothelial cell damage, promoting cerebral and other severe malaria illnesses.

### ICAM-1 Signaling

ICAM-1 mediates the sequestration of *P. falciparum* in the brain through the binding of PfEMP1 on the surface of IRBCs to endothelial cells (15, 16, 25). The sequestration of parasites in vascular capillaries of the brain induces inflammatory responses, resulting in the infiltration of cytotoxic effector cells and endothelial barrier damage. These processes have been implicated in the development of cerebral malaria, but the experimental evidence is controversial. An earlier study has reported that interaction with ICAM-1 contributes to increased serum TNF-α in cerebral malaria model of *P. berghei* ANKA-infected mice and that ICAM-1 deficient mice survive >15 days compared to 6–8 days in wild type (WT) mice (170). However, a later study reported that ICAM-1 is dispensable for cerebral malaria pathogenesis (171). NK cells cross-talk with myeloid cells through LFA-1 binding to ICAM-1, producing IFN-γ (172). Similarly, T cells are also known to interact with endothelial cells through LFA-1 mediated binding to ICAM-1, producing IFN-γ. Studies have shown that, upon binding to its ligands, ICAM-1 is activated by phosphorylation, inducing Src-PI3K-Akt, ${\text{Ca}}_{2}^{+}$, Rho and MAPK signaling (121, 122) (Figure 2). It is likely that these signals contribute to immune responses to malaria; thus far this aspect has not been examined.

## Innate Immune Responses to Malaria

### Innate Immune Responses at the Liver Stage Infection

In malaria, like in most pathogenic infections, the innate immune system functions as the first line of defense by controlling parasite growth and regulating the development of adaptive immunity (14, 75–77). As discussed in section Liver Stage Parasite Sensing, during the liver stage malaria infection, parasite-infected hepatocytes produce type I IFNs through cytosolic sensing of RNA. This cytokine response contributes to the killing of parasite-infected hepatocytes by NKT cells, exposing parasite components. The antigen-presenting cells, primarily DCs and inflammatory Mφs, can potentially recognize the exposed parasite factors. In addition, sporozoites injected by infected mosquitoes that could not enter the blood circulation remain in dermis and die. In addition, some sporozoites that enter blood circulation may not invade hepatocytes and die. These dead parasites are likely sampled by DCs and inflammatory Mφs, leading to TLR- and inflammasome-mediated immune responses. The activated antigen-presenting cells have potential to modulate immune responses to the blood stage infection. However, because the parasite load in liver in natural infections is very low, the innate immune responses are likely to be also very low. Thus, the responses produced by antigen presenting cells against liver stage parasites may not exert a significant influence on immune responses induced by the blood stage parasites. However, in hyper endemic areas, repetitive infections in humans may induce immune tolerance in antigen-presenting cells that may modulate immunity to the blood stage infection to a certain extent.

### Innate Immune Responses at the Blood Stage Infection

During the blood stage infection, unlike the liver stage, parasites grow exponentially through repetitive erythrocytic cycles, rapidly accumulating the biomass. This leads to an efficient induction of innate immune responses. Early during the blood stage infection, DCs and Mφs are key first responders of the innate immune system. However, as noted above, Mφs upon internalization of infected erythrocytes, merozoites or hemozoin become immunosuppressive and dysfunctional (133–137); unable to secrete cytokines and chemokines. It appears that the primary role of Mφs during the early stages of blood stage malaria infection is to control parasite growth through phagocytic clearance. In contrast to Mφs, DCs efficiently produce cytokines and chemokines in response malaria parasites, and effectively interact with cells of the innate and adaptive immune system. Thus, DCs play key roles in the initiation and regulation of innate and adaptive immunity to malaria. Also, it should be noted that some subsets of Mφs having certain features of DCs, such as CD11c^+^ spleen red pulp Mφs and CD169^+^ inflammatory Mφs, produce cytokines (127, 133, 173).

As noted in section RNA, during the blood stage malaria infection, type I IFNs are the earliest (24 h postinfection) cytokines produced through the activation of TLR7-MyD88 and IRF7 signaling (174). Early type I IFN response is produced primarily by the spleen red pulp Mφs and pDCs in *P. chabaudi*-infected mice (106, 127). Two recent studies by using lethal malaria model of *P. yoelii* YM infection, in which parasites grow rapidly to attain peak parasitemia of ~80% by 6 days postinfection, have also showed TLR7-dependent peak levels of type I IFN production at 24–36 h postinfection (81, 128). This early cytokine response is mediated through the coordination of TLR7- and cytosolic sensing mechanisms: TLR7-MyD88-IRF7 signaling in endosomes, and DNA-cGAS-STING-TBK1-IRF3/IRF7 and RNA-MDA5-MAVS-TBK1-IRF3 signaling in the cytosol (81). It has been found that SOCS1 expressed in response to cytosolic nucleic acid sensor-mediated signaling significantly downregulates TLR7-mediated type I IFN response. As such, deficiency in SOCS1 results in markedly high levels of type I IFNs, providing resistance against high parasite burden-dependent lethality (81). Additionally, it has been shown that CD169^+^ Mφs activated through STING-mediated signaling migrate to bone marrow, where they interact with pDCs to induce type I IFN production through TLR7-MyD88 signaling (128). Interaction of classical DCs with pDCs is also important in pDCs producing cytokine responses to malaria parasites (38, 108). It appears that Mφs and neutrophils that internalize parasites undergo pyroptosis (111), exposing parasite components, which are taken up by classical DCs and CD169^+^ Mφs, and activated through STING-mediated cytosolic signaling. The early type I IFN response triggers the infiltration of immune cells to the blood that may subsequently localize to the spleen. In contrast, in *P. berghei* ANKA*-*infected mice, relatively high level of IFN-α was seen at 4 days postinfection with low or negligible IFN-α production during 1–3 days postinfection (175). Collectively, the above results indicate that different strains of malaria parasites differentially induce type I IFN. That is, the earliest type I IFN production (24–36 h postinfection) by *P. chabaudi* and *P. yoelii* is mediated mainly through parasite RNA-induced TLR7 signaling (81, 106, 128), whereas such RNA-mediated early type I IFN response is not readily apparent in *P. berghei* ANKA (175). The production of type I IFNs at later stages of *P. berghei* ANKA infection likely involves a different mechanism. In other studies, cGAS sensing of the AT-rich motifs of *P. falciparum* DNA in the cytosol could induce type I IFN production through the STING-TBK1-IRF3/IRF7 signaling pathway, independent of TLR9-MyD88 signaling (109, 110). This could be the mechanism through which *P. berghei* ANKA induces significant levels of type I IFN at 4 days pi (175).

Early type I IFN response to malaria infection contributes to the suppression of antiparasitic immunity and promotes cerebral and other severe malaria illnesses under certain situations. This is evident from the observations that deficiency in IFNαR in *P. yoelii* YM infection resulted in increased IFN-γ levels, significantly decreased parasitemia and resistant to parasite burden-dependent death (128). Also, in *P. berghei* ANKA-infected mice, which produce low levels of early type I IFN response but produce a significant amount at a somewhat later stage (4 days postinfection), deficiency in IFNαR or treatment with anti-IFNαR1 antibody resulted in increased IFN-γ production, increased number of IFN-γ-positive NK and CD4^+^ T cells in the spleen, and significantly decreased parasitemia and protection from cerebral malaria (175, 176). From these results, it is evident that type I IFNs suppress the production of IFN-γ and thus anti-parasitic function under certain conditions. By contrast, in a different situation, high levels of type I IFN production at early stages of *P. yoelii* YM infection promoted antiparasitic immunity. In this case, blocking of SOCS1, a suppressor of cytokine signaling 1, expression resulted in high levels of type I IFN production, leading to increased IFN-γ production and reduced parasitemia, protecting mice from parasite burden-dependent death (81). Consistent with the observations of the latter study (81), daily treatment of *P. berghei* ANKA-infected mice with recombinant IFN-α, which to a certain extent resembles a situation of high levels of type I IFN production at early stage of infection, also significantly increased IFN-γ production by splenic CD8^+^ T cells, markedly reducing parasitemia and preventing cerebral malaria (177). The results of these two studies suggest that type I IFNs promote IFN-γ-dependent anti-parasitic immunity, providing resistance against severe malaria. On the other hand, treatment of *P. berghei* ANKA-infected mice with recombinant IFN-β resulted in reduced TNF-α and IFN-γ production, decreased expression of ICAM-1 and CXCL9 in the brain, reduced CXCR3 expression by T cells and T cell infiltration to the brain, thereby significantly preventing cerebral malaria and increasing mice survival (178). Thus, type I IFNs play contrasting roles in malaria in a context-dependent manner and also dependent on type I IFN isomeric composition.

Compared to the results of two studies outlined above (175, 176), wherein deficiency in IFNαR contributed to increased IFN-γ response and decreased parasitemia in *P. berghei* ANKA-infected mice, Palomo *et al*. observed contrasting immune responses (179), although in all these studies mice were protected from cerebral malaria. Palomo et al. found that, compared to infected WT mice, *P. berghei* ANKA-infected mice deficient in IFNαR had similar parasitemia, reduced expression of IFN-γ and granzyme B by T cells, decreased expression of T cell-attracting chemokine CXCL9, and low infiltration of CXCR3^+^-expressing CD8 T cells to the brain. A notable difference in these studies is that the latter study used GFP knock-in transgenic parasite clone, which appeared to have slower growth rate than the WT parasites used in the former study. These differences may alter the dynamics of immune responses. Nevertheless, the results agree with the notion that type I IFNs play distinct roles under different malaria conditions.

Overall, the available data indicate that type I IFNs play disparate roles in malaria infection depending on the timing and amount of production, relative levels of IFN-α, IFN-β and perhaps other isomers, and compositions of cellular and cytokine milieu during the progression of infection, and parasite strains. A recent review provides a comprehensive and up-to-date account on the role and contrasting effects of type I IFNs in malaria (180). Type I IFNs are pleiotropic cytokines and as such, they induce a wide range of effects on innate and adaptive immune cells during various pathogenic infections, contributing to either protection against infection or pathogenesis (181–183). These differential effects are likely dependent on the levels of type I IFNs; low levels at early stages of infection mediate cell-mediated immunity, but high levels cause immunosuppression (182). Thus, it is not surprising that type I IFNs produced during malaria infection also induce a wide range of cellular effects involving a complex interplay between various cell types.

In addition to producing type I IFNs, DCs produce a wide range of pro-inflammatory cytokines, including TNF-α, IL-12, and IL-6, and chemokines, such as CXCL1, CXCL2, CCL2, CCL5, CXCL9, and CXCL10 in response to malaria parasites, and play crucial roles in malaria immunity and pathogenesis (34, 38, 108, 184–186). Type I IFNs prime DCs for efficient cytokine and chemokine production and activate NK, NKT, γδ T, and T cells to induce IFN-γ and other inflammatory responses (182). IL-12 produced by DCs activates NK cells to induce the production of IFN-γ, which promotes Th1 and effector T cell responses (187). The augmented production of IFN-γ contributes to an efficient parasitemia control by priming Mφs and neutrophils for increased phagocytic activity and thus parasite clearance (188, 189). IFN-γ also contributes to cerebral and other severe malaria clinical conditions under certain situations, such as parasite sequestering in vital organs (187, 188, 190). Chemokines, on the other hand, promote the recruitment of immune cells to mount effective cell-mediated anti-parasitic effects (191). However, these responses also contribute to severe pathology (37, 192, 193). By and large, the initial innate immune responses are mainly aimed at controlling parasite growth by potentiating antiparasitic cell-mediated immunity. However, since pro-inflammatory responses contribute to pathogenesis (14, 33–38), as infection progresses, the function of DCs switches from pro-inflammatory and Th1-inducing to anti-inflammatory and Th2-inducing phenotypes (39, 40, 194). Eventually, balanced pro- and anti-inflammatory and Th1/Th2 responses prevent pathogenesis and promote protective humoral immunity to malaria (39–42); imbalanced responses contribute to pathogenesis. Thus, innate immune responses contribute to either protective immunity or pathogenesis in a context dependent manner.

### TLR-MYD88 Signaling Prominently Contributes to Protective Immunity and Pathogenesis

DNA and RNA play prominent roles in mouse malaria immunity and pathogenesis; under certain conditions TLR2 and TLR4 also play important roles. As such, studies in various mouse models have demonstrated that TLR9, TLR7, TLR4, and TLR2 play important roles in malaria immunity and cerebral, placental and other severe malaria pathology (43, 44, 81, 102, 103, 106–108, 124, 128, 174, 195–201). Gene polymorphism studies in endemic areas that assessed the role of TLRs in malaria have linked TLR9, TLR4, and TLR2 to either susceptibility or resistance to malaria (202–214). While the involvement of TLR9, TLR4, and TLR2 in malaria immunity/pathology is evident from studies in both mouse malaria models and in humans (43, 106, 195, 197, 198, 200–203, 205, 210), thus far none of the studies in endemic areas have revealed the association of TLR7 with human malaria immunity/pathology. It is unclear whether studies in endemic areas that have assessed association of TLRs have investigated the role of TLR7 in malaria susceptibility/resistance. However, since *P. falciparum* RNA appears to have very low immunostimulatory activity, it is possible that TLR7 has either minor or no role in human malaria. Accumulated evidence indicates that TLRs prominently contribute malaria immunity and pathology, whereas other signaling mechanisms such as inflammasome and adherence-mediated signaling may play minor roles (172, 215).

The contribution of pro-inflammatory responses produced by the innate immune system to protective immunity and pathogenesis is primarily context dependent. In non-cytoadherent parasites, such as *P. yoelii* 17XNL strain and *P. chabaudi* that exhibit slow growth rates during the initial phase of infection, pro-inflammatory responses are protective by facilitating anti-parasitic effector function and cell-mediated immunity. In contrast, in the case of cytoadherent parasites, such as *P. berghei* ANKA that sequesters in brain, lungs, liver and adipose tissues, and *P. berghei* NK65 that sequesters in lungs and liver, pro-inflammatory responses are pathogenic by promoting cytotoxicity in effector cells, which cause organ damage. In this regard, the observations made in mouse models parallel those of vivax and falciparum malaria in humans. In *P. falciparum* infection, strong pro-inflammatory responses contribute to an effective control of parasitemia which otherwise results in fulminant infection (41, 42). However, despite this beneficial effect, pro-inflammatory responses promote effector cell function, which contributes to organ damage and severe illnesses. In contrast, in the case of *P. vivax*, which is relatively slow growing and does not cytoadhere except at low levels in lungs and the placenta (125), despite causing a number of clinical conditions, fatal illnesses are relatively rare compared to *P. falciparum*.

## Concluding Remarks

Malaria continues to be a major global health problem. In many endemic areas, parasites are becoming increasingly resistant to the currently widely used artemisinin-based combination drugs, which have been very effective in treating infection. Therefore, new drugs or other treatment strategies are urgently needed. Mass vaccination is the best strategy to prevent malaria. However, despite huge efforts during the past decades in many laboratories around the world, obtaining an efficacious vaccine suitable for mass vaccination remains challenging. It is thought that better understanding of molecular and cellular processes involved in the development of protective immunity and those that contribute to severe pathogenesis will be useful in designing an effective vaccine. During the past decades, a significant progress has been made in identifying the parasite factors and host receptors involved in the activation of the innate immune system and the associated cell signaling pathways. Significant progress has also been made in understanding how cellular activation and immune responses initiated upon parasite-host interactions influence subsequent development of antiparasitic immunity and contribute to severe pathogenesis.

## Author Contributions

All authors listed have made a substantial, direct and intellectual contribution to the work, and approved it for publication.

### Conflict of Interest Statement

The authors declare that the research was conducted in the absence of any commercial or financial relationships that could be construed as a potential conflict of interest.

## Abbreviations

IRBCs: infected red blood cells
DC: dendritic cell
Mφ: macrophage
NK cells: natural killer cells
NKT cells: NK T cells
GPI: glycosylphosphatidylinositol
PfEMP1: *P. falciparum* erythrocyte membrane protein 1
ICAM-1: intracellular adhesion molecule 1
VCAM-1: vascular adhesion molecule 1
LFA-1: lymphocyte function-associated antigen-1
EPCR: endothelial protein receptor
Th: helper T cells
IFNαR: type I IFN-α receptor
PAMPs: pathogen-associated molecular patterns
DAMPs: danger-associated molecular patterns
PRRs: pathogen recognition receptors
TLR: toll-like receptor
MyD88: myeloid differentiation factor 88
MAL: MyD88 adapter-like
IRAK: IL-1 receptor-associated kinase
TRAF6: tumor necrosis factor receptor-associated factor 6
TRIF: TIR (Toll/IL-1 receptor) domain-containing adaptor inducing IFN-β
TRAM: TRIF-related adaptor molecule
MAPK: mitogen-activated protein kinases
ERK: extracellular signal-regulated kinases
JNK: c-Jun N-terminal kinases
NF-κB: nuclear factor κB
TBK1: TRAF family member-associated NF-κB activator (TANK)-binding kinase 1
cGAS: cyclic GMP-AMP synthase
STING: stimulator of IFN genes
MDA5: melanoma differentiation-associated gene 5
MVAS: mitochondrial antiviral-signaling protein
IRF: IFN regulatory factor
AIM2: absent in melanoma 2
NOD: nucleotide-binding oligomerization domain
NLR: (NOD)-like receptor
LLR: leucine-rich repeat kinase-like protein
NLRP: NOD- LRR- and pyrin domain-containing protein.
